# Non-consensual Sex Among Japanese Women in the COVID-19 Pandemic: A Large-Scale Nationwide Survey-Based Study

**DOI:** 10.7759/cureus.70873

**Published:** 2024-10-05

**Authors:** Tomoya Suzuki, Yasuhiro Kotera, Akihiko Ozaki, Tetsuya Tanimoto, Divya Bhandari, Sayaka Horiuchi, Takahiro Tabuchi

**Affiliations:** 1 Pediatric Medicine, Okinawa Prefectural Chubu Hospital, Okinawa, JPN; 2 Faculty of Medicine and Health Sciences, University of Nottingham, Nottingham, GBR; 3 Department of Breast and Thyroid Surgery, Jyoban Hospital of Tokiwa Foundation, Iwaki, JPN; 4 Internal Medicine, Navitas Clinic Kawasaki, Kawasaki, JPN; 5 Research, Medical Governance Research Institute, Minato-ku, Tokyo, JPN; 6 Graduate Faculty of Interdisciplinary Research Faculty of Medicine Basic Medicine (Epidemiology/Environmental Medicine), University of Yamanashi, Yamanashi, JPN; 7 Division of Epidemiology, School of Public Health, Tohoku University Graduate School of Medicine, Sendai, JPN

**Keywords:** covid-19 outbreak, non-consensual sexual act, sexual abuse trauma, sexual distress, sexual problems

## Abstract

Introduction and background

Non-consensual sex, including rape and sexual assault, has been a global concern and may have been influenced by the COVID-19 pandemic. However, information on this topic is limited. Therefore, our objective was to survey the incidence rate of non-consensual sex among Japanese women aged 15-79 years between April and September 2020, during the early stages of the COVID-19 pandemic in Japan.

Materials and methods

We utilized data from a sample of approximately 2.2 million individuals who participated in a web-based self-reported questionnaire survey from a nationwide, cross-sectional internet survey conducted in Japan between August and September 2020. Sampling weights were applied to calculate national estimates, and multivariable logistic regression was performed to identify factors associated with non-consensual sex. Data were extracted from a web-based, self-administered survey of approximately 2.2 million individuals.

Results

The study examined the incidence of non-consensual sex among 12,809 women with valid responses, finding an overall rate of 1.1% across all participants. Higher rates were observed among women aged 20-29 (2.4%) and employed women (1.5%) compared to unemployed women (0.7%). No significant difference was noted based on living areas. Increased FCV-19S scores, worsening or improving mental health before COVID-19, suicidal thoughts, and feelings of isolation were all linked to higher incidence rates. Non-payment of salary and lack of money for necessities also correlated with higher rates. Key risk factors included age 15-19 or 20-29, employment, financial instability, suicidal thoughts, and isolation. Notably, 20% of women aged 15-19 reported suicidal ideation.

Conclusions

This study underscores the critical need for mental and financial support for young women, highlighting the importance of early intervention for economically vulnerable groups. Comprehensive education on sexual consent is essential, especially during societal upheavals like the COVID-19 pandemic, to prevent non-consensual sex and support affected individuals.

## Introduction

Non-consensual sex refers to engaging in sexual behavior without obtaining consent to touch, observe, or perform sexual acts involving private body parts that exceed the boundaries of another person’s body or mind [1,2]. Non-consensual sex can take many forms, including rape, transactional sex, cross-generational sex, unwanted touch, and molestation. In the European Union, between 45% and 55% of women have experienced sexual harassment since the age of 15 [3]. Previous research has identified common factors associated with victims, including ages between 10 and 30 years, mental vulnerability, and financial hardship [4]. Women with these characteristics are at a higher risk of experiencing non-consensual sex compared to those without these traits [4]. Furthermore, women with these characteristics often find it challenging to disclose incidents of non-consensual sex to others, making it difficult to address these issues [5].

Of note, non-consensual sex is reported to increase due to disruptions in social conditions, such as natural disasters and worsened economic conditions. For instance, incidents of non-consensual sex against women dramatically increased during times of economic and psychological turmoil, such as the 2005 Hurricane Katrina in the United States and the 2011 Great East Japan Earthquake [6,7]. These studies have shown that sexual violence rates often rise after large-scale crises, such as natural disasters, due to social disruption, economic instability, and isolation. The COVID-19 pandemic has worsened these factors, potentially increasing the incidence of non-consensual sex. Therefore, it is crucial to investigate the incidence rate of non-consensual sex after the COVID-19 pandemic.

At the onset of the COVID-19 pandemic, it was reported that 243 million girls and women aged 15-49 years had been subjected to non-consensual sex worldwide [8]. This was attributed to the increase in the mandatory stay-at-home measure due to lockdowns, resulting in reduced access to social support from teachers, friends, and caregivers [9]. Awareness of the status of non-consensual sex during the COVID-19 pandemic has grown in many countries [10]. However, the frequency and characteristics of non-consensual sex remain to be elucidated, not only after but also before the COVID-19 pandemic in Japan to date, partly due to traditional social norms that discourage open discussions about sexual issues [11].

Our study aimed to report the incidence of non-consensual behavior during the early stages of the COVID-19 pandemic in Japan. Additionally, we explored potential risk factors such as mental health conditions, economic changes, concerns about the lockdown, and anxiety about the disease.

## Materials and methods

Context

This cross-sectional study was conducted among a sample of approximately 2.2 million individuals from the general public who participated in a web-based self-reported questionnaire survey for the Japan COVID-19 and Society Internet Survey (JACSIS) project [12]. The survey was administered by the well-established internet research agency Rakuten Insight, Inc., which has been utilized in prior research studies [12,13]. Participants were recruited, and data were collected from August 2020 to September 30, 2020. The prefectures where the selected individuals lived represented all prefectures in Japan, as indicated in previous studies [14].

Patient and public involvement

No patients were involved in this study.

Participants

The survey invitation was extended to 28,000 individuals out of 2.2 million registered with the survey agency Rakuten Insight, Inc. We selected study participants through random sampling using a computer algorithm.

Analysis subjects

Our analysis included respondents who self-identified as women in the questionnaire. Among all the survey respondents totaling 28,000, the number of valid female participants for analysis was 12,809.

Outcome variable: Experience of non-consensual sex

The focus of this study was to examine the incidence of non-consensual sex occurring between April 2020 and September 2020. Participants recruited from August to September 2020 were not questioned about specific details but were instead asked to respond with a simple “yes” or “no” to indicate whether they had experienced non-consensual sex during April 2020 to September 2020. We did not explicitly seek a specific definition of non-consensual sexual activity, which may vary by generations or community cultures (e.g., workplace community, religious community).

Exposure variables

Sociodemographic Characteristics

As independent variables, demographics were included, encompassing gender, age groups (15-19, 20-29, 30-39, 40-49, 50-59, 60-69, 70-79 years), marital status (unmarried, married and widowed/separated), having children (none or more than one), educational attainment (high school educated, college-educated or higher), employment status (any type of employment, unemployed), household income level, which was calculated as dividing the household income by the square root of household size (categorized by the tertials of household equivalent income (low, <JPY2.5 million/US$25 000/£16 667; intermediate, JPY2.5-JPY4.3 million/US$25 000-US$43 000/£16 667-£28 667; high, >JPY4.3 million/<US$43 000/<£28 667; unknown/declined to answer)), and smoking status (non-smoker or current smoker) [12].

Living Area Classification

Regarding living regions, all 47 prefectures were divided into the following three areas based on the date of the Declaration of the State of Emergency (DSE area) [15,16]. “Designated as DSE on April 7, 2020," “Designated as DSE on April 16, 2020," and "Others," “Specific alert designated on April 7, 2020” included Tokyo, Kanagawa, Chiba, Saitama, Osaka, Hyogo, and Fukuoka. “Specific alert designated on April 16, 2020” included Hokkaido, Ibaraki, Ishikawa, Gifu, Aichi, and Kyoto. “Others” comprised the remaining 34 prefectures. These categorizations were determined by the Japanese government using three indicators: the cumulative number of infected people, epidemiological trend, medical capacity, and surveillance system [15]. During the target period, people living in the relevant prefectures were advised to avoid unnecessary outings, limit the use of entertainment facilities, and adhere to restrictions on economic activities [17].

Fear of COVID-19 Scale

The Japanese version of the Fear of COVID-19 Scale (FCV-19S) was used to assess participants’ fear of COVID-19 infection [18]. This instrument comprises seven items rated on a 5-point scale (1 = “strongly disagree”; 5 = “strongly agree”). Consequently, the total score ranged from 7 to 35, with a higher score indicating a greater fear of COVID-19. Satisfactory internal consistency and validation of the scale were confirmed in the original seven-item scale (α = 0.82) [19].

Personal Health and Economic Statuses

Based on previous research, an examination was conducted to determine whether health and economic statuses were associated with non-consensual sex [4]. The health-related questions included the following: self-rated health (good or other than good), “Any change in mental state in the last month compared to before January 2020?” (worse, no change, getting better, I do not know),” suicidal thoughts (experience since before the COVID-19 pandemic, first experienced during the COVID-19 pandemic, or never), desire not to talk to anyone due to worries (yes or no), feeling isolated (yes or no), any cancellation of a family gathering due to the COVID-19 pandemic (yes or no), non-payment of salary (experience since before the COVID-19 pandemic, first experience during the COVID-19 pandemic, never), and lack of money to buy necessities of life (experience since before the COVID-19 pandemic, first experience during the COVID-19 pandemic, or never).

Statistical procedure

To achieve the purpose of identifying the incidence of non-consensual sexual activity and its associated factors during the COVID-19 outbreak, two types of analysis methods were employed.

First, we calculated the incidence of non-consensual sex by dividing the sample into two groups: those who responded 'yes' to experiencing non-consensual sex and those who did not. We then used a chi-square test to compare the distribution of independent variables (e.g., age, employment status) between these two groups. The chi-square test is suitable for categorical data and assesses whether there are significant differences in the frequency distribution of these variables between the groups. This test helps determine if the observed frequencies deviate from what would be expected by chance alone, thereby providing insights into potential associations between non-consensual sex and various independent variables. The number, percentage, and p-value of each variable are presented in Materials and Methods.

Second, a multivariable logistic regression analysis was conducted to identify potential predictive factors in the occurrence of non-consensual sex. This analysis encompassed all the aforementioned exposure variables, and odds ratios with 95% confidence intervals (CIs) were estimated. All exposure variables were included in the multivariable model due to their potential clinical and theoretical relevance, regardless of statistical significance. This approach helps ensure accurate risk assessment by accounting for possible confounders, as excluding variables could overlook important associations. As a sub-analysis, the incidence of suicidal ideation by age was also calculated.

Statistical significance was defined as p<0.05. The data were analyzed using STATA V.16.1 (Stata Corp., College Station, Texas, USA).

This article was previously posted to the medRxiv preprint server on February 18, 2024.

## Results

The total number of respondents, including men and women, was 28,000. Out of these, we excluded 2,518 participants who provided invalid responses (e.g., using the same number for all survey items; incomplete responses) and 12,673 men. Consequently, the final analysis sample consisted of 12,809 women, whose ages ranged from 15 to 79 years old.

Table 1 shows the characteristics of the weighted proportion of non-consensual sex among all participants. The overall weighted incidence rate for non-consensual sex during the five-month period (April to September 2020) was 1.1%. The incidence rate of non-consensual sex was higher among individuals aged 15-19 years (1.1%) and 20-29 years (2.4%), which exceeded the rates in other age groups. Women with jobs (1.5%) had a higher incidence rate than those without jobs (0.7%).

**Table 1:** Demographics and descriptive statistics of weighted women participants during the five-month from April to September 2020 †Classification of the Fear of COVID-19 scale scores refers to previous studies [19]. All estimates account for survey weights.

Variable	Non-consensual sex April-Sept 2020 total (n=12,809)	
	Yes	No
	N (%)	N (%)
Total	138 (1.1)	12,671 (98.9)
Age		
15–19	8.0 (1.1)	707 (98.9)
20–29	37 (2.4)	1543 (97.7)
30–39	38 (2.0)	1845 (98.0)
40–49	25 (1.0)	2395 (98.9)
50–59	15 (0.7)	2097 (99.3)
60–69	15 (0.7)	2127 (99.3)
70–79	1.0 (0.1)	1956 (99.9)
Marital status		
Married	88 (1.1)	7929 (98.9)
Never married	29 (0.9)	3276 (99.1)
Widowed or separated	20 (1.4)	1467 (98.7)
Having children		
No children	56 (0.9)	6006 (99.1)
At least one or more	99 (1.5)	6648 (98.5)
Educational attainment		
High school or lower	54 (1.0)	4261 (99.0)
College/university or graduate school	83 (1.2)	7136 (98.9)
Employment status		
Unemployed	43 (0.7)	6259 (99.3)
In employment	95 (1.5)	6412 (98.5)
Smoking status		
Non-smoker	117 (1.0)	11,404 (99.0)
Current smoker	20 (1.6)	1268 (98.4)
Household income (million JPY)		
Low	53 (1.7)	3144 (98.3)
Moderate	43 (1.3)	3226 (98.7)
High	26 (1.0)	2669 (99.0)
Unknown	14 (0.4)	3632 (99.6)
Region		
Other	86 (1.3)	6628 (98.7)
DSE (April 16)	16 (0.8)	2117 (99.2)
DSE (April 7)	31 (0.9)	3568 (99.1)
Fear of COVID-19^†^		
7–15	41 (1.2)	3402 (98.8)
16–20	26 (0.6)	4173 (99.4)
21–25	34 (1.0)	3405 (99.0)
26–35	37 (2.1)	1691 (97.9)
Self-rated health		
Other than good	94 (1.3)	7349 (98.7)
Good	44 (0.8)	5322 (99.2)
Mental state change in the last month compared to before January 2020		
Worse	59 (2.2)	2662 (97.8)
None	56 (0.6)	8998 (99.4)
Better	19 (3.3)	547 (96.7)
Unknown	4 (0.7)	500 (99.3)
Suicidal thoughts		
Yes, since before COVID-19	43 (4.0)	1039 (95.1)
First experience	20 (3.5)	539 (96.5)
Never	75 (0.7)	11,093 (99.3)
Desire not to talk to anyone due to worries		
Yes	76 (4.0)	2487 (97.0)
No	62 (0.6)	10,184 (99.4)
Feeling isolated		
Yes	74 (3.7)	1925 (96.3)
No	64 (0.6)	10,747 (99.4)
Cancellation of a family gathering due to the pandemic		
Yes	10 (0.6)	1730 (99.4)
No	127 (1.2)	10,940 (98.9)
Non-payment of salary		
Yes, since before COVID-19	15 (10.3)	133 (89.7)
First experience	8 (10.4)	67 (89.6)
Never	115 (0.9)	12,471 (99.1)
Lack of money to buy necessities		
Yes, since before COVID-19	35 (4.8)	692 (95.2)
First experience	22 (6.1)	340 (94.0)
Never	81 (0.7)	11,639 (99.3)

There was no significant difference in the incidence rate of non-consensual sex among those living in areas designated as DSE (0.9% on April 7, 2020, 0.8% on April 16, 2020, and 1.3% others). A higher FCV-19S (2.1% [score from 26 to 35]) was associated with a higher incidence rate compared to other scores (1.2% [score from 7 to 15], 0.6% [score from 16 to 20], 1.0% [score from 21 to 25]).

The incidence rate of participants who reported feeling “worse” (2.2%) or “getting better” (3.3%) before the COVID-19 pandemic was higher than those who reported “no change” (0.6%) and “I do not know” (0.7%). Participants who had suicidal thoughts (4.0% since before COVID-19, 3.5% first experience) had a higher incidence rate than those who had never experienced them (0.7%). Participants who reported feeling isolated (3.7%) had a higher incidence rate than those who had never felt isolated (0.6%). Additionally, participants who had experienced non-payment of salary (10.3% since before COVID-19, 10.4% first experience) had a higher incidence rate than those who had never experienced it (0.9%). Similarly, participants who had experienced a lack of money to buy necessities (4.8% since before COVID-19, 6.1% first experience) had a higher incidence rate than those who had never experienced it (0.7%).

Table 2 shows the factors associated with non-consensual sex among participants. Participants aged 15 to 19 years and those aged 20 to 29 years had higher adjusted odds of experiencing non-consensual sex compared to participants in other aging groups. (aged 15-19 years old: OR 4.74, 95% CI 1.37-16.4, aged 20-29 years old: OR 3.20, 95% CI 1.09-9.38). Employed women participants were associated with higher adjusted odds of experiencing non-consensual sex than unemployed participants (OR 1.68, 95% CI 1.36-2.07).

**Table 2:** Factors associated with non-consensual sex among weighted participants during the-five-month from April to September 2020 Weighted incidence (%) represents the actual number of cases (shown as a number) and the proportion of those cases as a percentage (%). *A p-value of less than 0.05 was considered statistically significant.

	Weighted sample	Weighted incidence (%)	Crude Odds (95% CI)	Adjusted rate % (95% CI)	Adjusted odds ratio (95% CI)	P-value
Total	12,809	138 (1.1)	-	-	-	-
Age						
15–19	715	8.0 (1.1)	2.26 (1.29, 3.94)	3.4 (1.3, 5.5)	4.74 (1.37, 16.4)	0.014*
20–29	1,580	37.1 (2.4)	1.74 (1.07, 2.81)	2.4 (1.3, 3.4)	3.2 (1.09, 9.38)	0.034*
30–39	1,883	37.7 (2.0)	1.37 (0.84, 2.23)	1.5 (0.9, 2.1)	1.88 (0.63, 5.55)	0.256
40–49	2,420	24.5 (1.0)	Reference	0.8 (0.3, 1.3)	Reference	-
50–59	2,112	14.5 (0.7)	0.43 (0.22, 0.83)	0.8 (0.5, 1.0)	0.90 (0.37, 2.19)	0.822
60–69	2,142	14.8 (0.7)	0.49 (0.26, 0.92)	1.1 (0.6, 1.7)	1.35 (0.45, 4.06)	0.585
70–79	1,957	1.0 (0.1)	0.23 (0.10, 0.55)	0.05 (0.0, 0.1)	0.05 (0.02, 0.19)	<0.001*
Marital status						
Married	8,017	88.3 (1.2)	Reference	1.6 (1.2, 2.0)	Reference	-
Never married	3,304	29.0 (0.8)	1.35 (0.96, 1.90)	0.5 (0.1, 0.8)	0.26 (0.10, 0.70)	0.007*
Widowed or separated	1,487	20.3 (1.2)	0.83 (0.48, 1.43)	1.5 (0.8, 2.1)	0.92 (0.69, 1.24)	0.599
Having children						
No children	6,302	42.8 (0.7)	Reference	0.8 (0.7, 0.9)	Reference	-
One or more	6,507	94.9 (1.4)	1.60 (1.15, 2.22)	1.2 (1.1, 1.4)	1.58 (1.23, 2.02)	<0.001*
Educational attainment						
College/university or graduate school	7,219	83.3 (1.0)	Reference	0.9 (0.8, 0.9)	Reference	-
High school or less	5,590	54.4 (1.3)	0.76 (0.53, 1.08)	1.3 (1.2, 1.3)	0.66 (0.57, 0.75)	<0.001*
Employment status						
Unemployed	6,237	43.2 (0.7)	Reference	0.8 (0.7, 0.9)	Reference	-
Employed	6,572	94.5 (1.5)	1.701 (1.23, 2.37)	1.3 (1.2, 1.4)	1.68 (1.36, 2.07)	<0.001*
Smoking status						
Non-smoker	11,521	117.4 (1.0)	Reference	1.1 (1.0,1.2)	Reference	-
Current smoker	1,288	20.3 (1.6)	1.63 (1.05, 2.53)	1.0 (0.3, 1.6)	0.89 (0.39, 2.04) 1.08 (0.67, 3.83)	0.779
Household income (million JPY)						
Low	3,579	57.2 (1.5)	Reference	1.4 (0.9, 1.8)	Reference	-
Moderate	3,271	49.3 (1.6)	1.28 (0.68, 1.56)	1.4 (1.4, 1.5)	1.06 (0.77, 1.46)	0.715
High	2,313	16.6 (0.6)	1.01 (0.66, 1.53)	0.8 (0.3, 1.3)	0.57 (0.21, 1.56)	0.275
Unknown	3,647	14.5 (0.5)	0.467 (0.28, 0.79)	0.4 (0.2, 0.7)	0.29 (0.11, 0.75)	0.011*
Region						
Other	6,715	86.4 (2.0)	Reference	1.1 (1.0, 1.1)	Reference	-
DES (April 13)	2,133	16.3 (0.8)	1.12 (0.70, 1.78)	1.1 (1.1, 1.2)	1.07 (0.99, 1.15)	0.100
DES (April 7)	3,599	31.2 (0.5)	1.03 (0.72, 1.48)	1.0 (1.0, 1.1)	0.94 (0.68, 1.24)	0.261
Fear of COVID-19						
7–15	3,443	40.9 (1.2)	Reference	1.4 (1.1, 1.7)	Reference	-
16–20	4,199	25.9 (0.6)	0.781 (0.50, 1.21)	0.7 (0.4, 1.0)	0.45 (0.23, 0.88)	0.019*
21–25	3,439	34.3 (0.9)	0.832 (0.53, 1.30)	0.9 (0.9, 1.0)	0.62 (0.47, 0.81)	0.001*
26–35	1,728	36.5 (2.3)	2.00 (1.28, 3.11)	1.4 (1.3, 1.5)	1.01 (0.84, 1.21)	0.927
Self-rated health						
Good	5,366	43.8 (0.7)	Reference	0.9 (0.8, 1.1)	Reference	-
Other than good	7,443	93.9 (1.5)	1.39 (1.01, 1.91)	1.2 (1.1, 1.2)	1.25 (0.98, 1.48)	0.069
Mental state change in the last month compared to before January 2020						
Worse	9,054	56.0 (0.6)	Reference	0.9 (0.7, 1.2)	Reference	-
None	2,686	59.3 (2.2)	3.14 (2.19, 4.49)	1.1 (0.8, 1.5)	1.25 (0.67, 2.33)	0.480
Better	566	18.8 (3.0)	6.53 (4.15, 10.30)	1.8 (1.4, 2.3)	2.11 (1.39, 3.18)	<0.001*
Unknown	504	3.5 (0.8)	1.55 (0.622, 3.88)	0.8 (0.7, 0.8)	0.80 (0.53, 1.20)	0.286
Suicidal thoughts						
Yes, since before COVID-19	11,169	75.2 (0.7)	Reference	0.9 (0.8, 1.0)	Reference	-
First experience	1,082	42.8 (3.9)	5.85 (4.06, 8.43)	1.6 (1.4, 1.8)	1.85 (1.50, 2.27)	<0.001*
Never	558	19.7 (4.3)	7.66 (4.85, 12.01)	1.1 (0.7, 1.5)	1.25 (0.79, 1.97)	0.337
Desire not to talk to anyone due to worries						
Yes	10,246	61.5 (0.6)	Reference	0.8 (0.7, 0.9)	Reference	-
No	2,563	76.2 (3.2)	7.27 (5.24, 10.08)	1.5 (1.3, 1.7)	1.98 (1.56, 2.51)	<0.001*
Feeling isolated						
Yes	10,811	64.1 (0.6)	Reference	0.8 (0.3, 1.2)	Reference	-
No	1,988	73.6 (3.4)	8.35 (6.02, 11.60)	1.8 (0.6, 2.9)	2.52 (0.70, 9.04)	0.156
Cancellation of a family gathering due to the pandemic						
Yes	1,741	83.3 (1.0)	Reference	0.5 (0.1, 0.9) 0.8 (8.9, 15.6)	Reference	-
No	11,068	54.4 (1.3)	1.035 (0.64, 1.68)	1.2 (1.1, 1.3)	2.58 (1.07, 6.25)	0.035*
Non-payment of salary						
Yes, since before COVID-19	12,586	7.8 (7.3)	Reference	1.0 (0.9, 1.0)	Reference	-
First experience	148	114.6 (0.9)	12.15 (7.02, 21.01)	3.9 (2.9, 4.9)	4.88 (3.42, 6.97)	<0.001*
Never	75	15.3 (10.4)	15.13 (8.40, 27.28)	1.9 (1.1, 2.8)	2.17 (1.36, 3.48)	0.001*
Lack of money to buy necessities						
Yes, since before COVID-19	11,720	80.5 (0.7)	Reference	0.8 (0.7, 0.9)	Reference	-
First experience	727	34.9 (5.7)	6.97 (4.68, 10.38)	2.2 (1.9, 2.6)	2.94 (2.27, 3.81)	<0.001*
Never	362	22.2 (6.1)	7.62 (4.74, 12.25)	1.9 (1.1, 2.8)	2.50 (1.76, 3.55)	<0.001*

Participants who did not receive salaries or compensations were more likely to experience non-consensual sex than those who did: experienced since before COVID-19 (OR 4.88, 95% CI 3.42-6.97); first experience during COVID-19 (OR 2.17, 95% CI 1.36-3.48). Similarly, participants who could not afford to buy life necessities were more likely to experience non-consensual sex than the others (OR 2.5, 95% CI 1.76-3.55).

Lastly, participants who had suicidal thoughts were more likely to experience non-consensual sex than those who did not (since before COVID-19: OR 1.85, 95% CI 1.50-2.27). Participants who reported feeling isolated were more likely to experience non-consensual sex than those who did not (OR 1.98, 95% CI 1.56-2.51). The result of the sub-analysis showed suicidal ideation was present in 20% of all women aged between 15 and 19 years.

**Figure 1:**
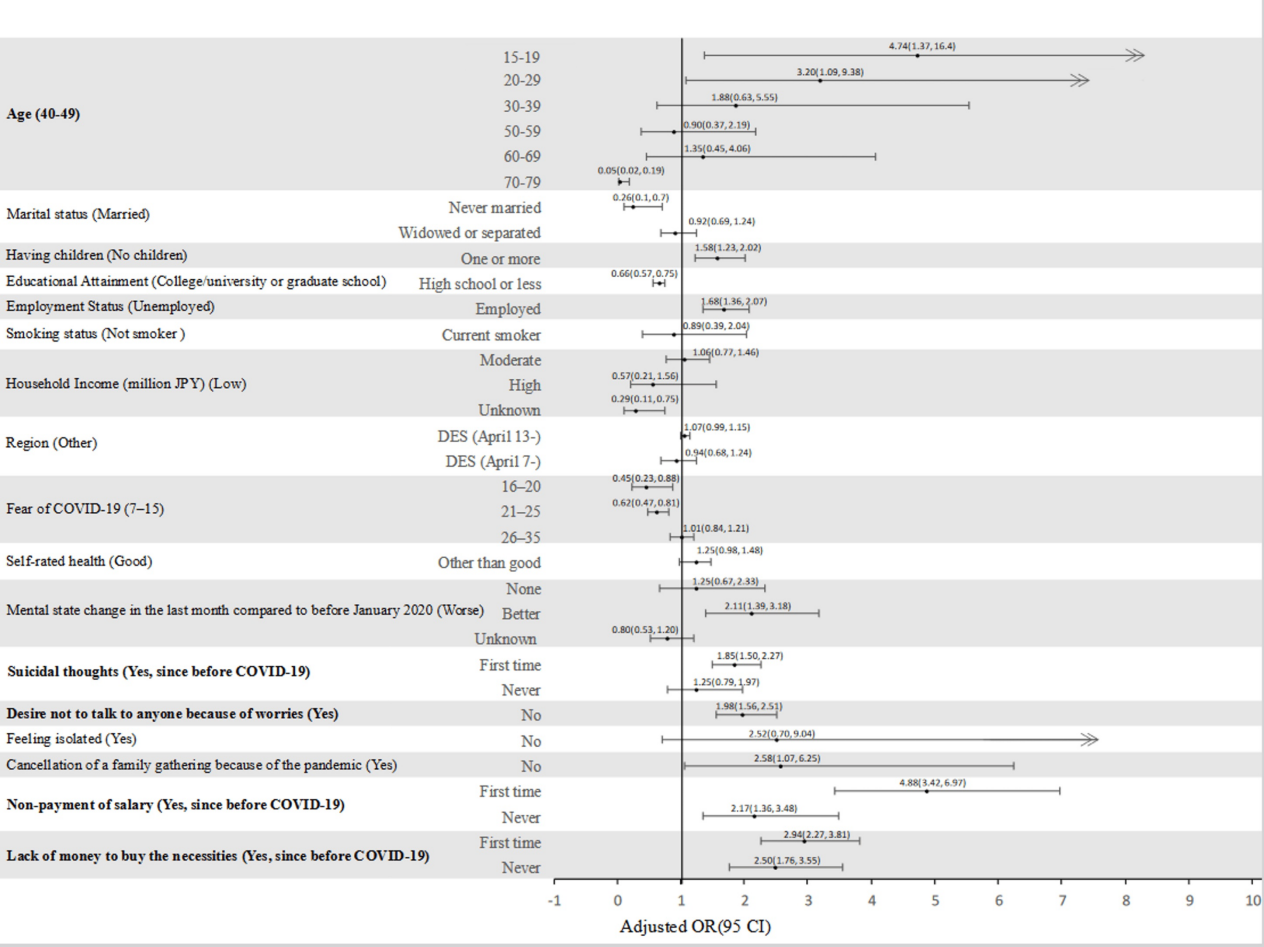
Forest plot illustrating the incidence rate of non-consensual sex

Figure 1 displays the forest plot, highlighting significant positive factors associated with non-consensual sex. The plot indicates that individuals in the age group "15-29 years old" and those experiencing worsened mental or economic status have an increased likelihood of encountering non-consensual sex. The plot visually represents these factors along with their effect sizes and confidence intervals, illustrating the association between these variables and the risk of non-consensual sex.

## Discussion

This study assessed the incidence of non-consensual sex in Japan during the COVID-19 pandemic and explored related factors while applying weighting to address biases from online participation. The rate of non-consensual sex reported by participants was 1.1% over a five-month period from April to September 2020. Key factors associated with non-consensual sex included age (15 to 29 years), employment status, worsening mental health, suicidal thoughts, isolation, a desire to avoid communication, and challenging economic conditions. These findings underscore the need for targeted interventions to address mental health and economic challenges exacerbated by the pandemic.

In Japan, research related to sexual matters is limited. We believe this limitation stems from the fact that sexual topics are often considered taboo in Japanese education institutions, and sexual education in Japan lags behind that in Western countries [20-22]. Additionally, the understanding of obtaining sexual consent, which is essential in all sexual activities, lags behind compared to countries that are known to be advanced in sex education in some European countries [11,20,23]. For instance, the Netherlands had sex education compulsory from elementary school, and Denmark was one of the European countries that criminalized non-consensual sexual acts in 2020 at the time of our survey. Despite the efforts in Europe, conducting such a comprehensive study on a large scale was of significant importance as it ventured into uncharted territory in Japan.

The global concern about increased sexual violence against women noted around April 2020 during the early COVID-19 outbreak points to a critical issue. However, the limited specific incidence data [10,24] highlights the need for more detailed research. This gap in data underscores the importance of improving data collection and developing targeted interventions to address sexual violence during public health emergencies [10,24]. While some countries reported an uptick in sexual violence cases (e.g., South Africa, Bangladesh [10,25]), others reported a significant decrease after implementing lockdown measures, as seen in Australia and Canada [26,27]. In Japan, the available data from the police included two key pieces of information: the reported incidence rate of sexual violence among women stood at 6.9% [28], and the number of sexual assault victims seeking consultations increased by approximately 1.2 times in 2020 compared to 2019 [29,30]. However, the incidence rate had not been thoroughly investigated during the COVID-19 pandemic, resulting in a scarcity of information regarding the incidence of sexual violence against women [31]. It is important to note that our study did not have access to a comparative dataset from a different time point to evaluate the percentage change in the incidence of sexual violence. Nevertheless, we were able to find fundamental data concerning non-consensual sex for the first time in Japan.

Approximately 80% of the women who reported experiencing non-consensual sex fell within the age range of 15 to 49 years old. Notably, the incidence of reported non-consensual sex dramatically decreased among women aged 50 to 79 years. There was a significant increase in the risk of non-consensual sex within the age group of 15 to 29 years. This pattern is consistent with reports from the United States and Japan, where individuals in their late teens to early thirties are identified as the age group at the highest risk of experiencing rape [28,32].

It is crucial to recognize that young women who have been victims of sexual assault are also more likely to experience lifetime suicide attempts and post-traumatic stress symptoms. Specifically, research indicates that teenage sexual trauma is strongly correlated with suicide attempts [33-36]. Additionally, our sub-analysis results revealed that suicidal ideation was present in 20% of all women aged between 15 and 19 years. Based on the aforementioned findings and considerations, we firmly believe that measures to protect the younger generation are imperative [37]. As mentioned previously, Japan falls behind in comprehensive sexual education for the younger generation. We contend that it is necessary to incorporate detailed explanations of the factors and circumstances that lead to unwanted sexual experiences involving non-consensual sex into the Japanese school curriculum.

Concretely, employed participants were more likely to be in contact with others than those who were unemployed. This time, we have not specifically investigated the locations where non-consensual sex has occurred. Therefore, it can be said that employed women may become victims of non-consensual sex, potentially by someone within their workplace or close social circle. Indeed, reports from both Japan and the United States have indicated that non-consensual sex is most frequently perpetrated by individuals known to the victims [28,38]. Employed people have an extra circle of known individuals from their workplace compared to unemployed people.

Intriguingly, women who did not receive a salary and lacked the financial means to purchase necessities were at an elevated risk of experiencing non-consensual sex. While this appears to be the opposite of the above finding suggesting that being employed could be associated with higher adjusted odds of experiencing non-consensual sex, this aligns with previous studies showing a connection between deteriorating economic conditions and an increased incidence of sexual assault that have established a connection between deteriorating economic conditions and an increased incidence of sexual assault [39,40], which is consistent with our findings. There are two possible explanations for this association: First, worsening personal economic circumstances may lead women to engage in occupations with a higher risk of non-consensual sex, such as working in the sex industry. Second, women in precarious financial situations often experience psychological pressure to secure and maintain employment [40]. Under such pressure, they may become psychologically vulnerable to sexual coercion from their employers in exchange for job security [40]. Future research should explore people in these circumstances in depth in better-underexplored areas in Japan.

While 66% of the participants lived in areas under DSE, our study did not find a significant association between non-consensual sex and living in the DSE area. However, we did identify a significant correlation between experiencing a moderate level of fear related to the COVID-19 pandemic (scoring 16-25 points) and instances of non-consensual sex. This suggests that participants who stayed at home more frequently might have become more vulnerable to non-consensual sexual experiences by their partners. In fact, non-consensual sex was more frequently reported as being perpetrated by partners during the COVID-19 pandemic, and there was an observed increase in the number of domestic problems during this period [29].

Based on the considerations outlined above, there is a heightened risk of non-consensual sexual activity occurring during the COVID-19 pandemic, particularly among young, impoverished women. Consequently, it is of utmost importance to identify and provide economic and psychosocial support to these vulnerable individuals during times of social disruption. Furthermore, a notable deficiency in the current landscape of Japan is the absence of comprehensive education regarding the concept of sexual consent. Specifically, there is an urgent need for thorough instruction emphasizing the importance of men obtaining consent from women before engaging in any sexual activities [41]. This education should be disseminated at both the local and national levels, as it may necessitate a societal shift in awareness at all levels.

In a noteworthy development, on June 16, 2023, Japan amended the legal term “forced sexual intercourse crime” to “non-consensual sexual intercourse crime," explicitly stipulating that any sexual activity without consent is now considered a criminal offense [42]. We anticipate that this alteration will support victims who have endured their suffering in silence and were unable to report their experiences.

Limitations

This study has several limitations. First, as it is based on a web-based survey, the participants may not represent the general population. Specifically, individuals with low digital literacy may have been excluded. To address this, a weighted analysis was performed to minimize differences in demographic, economic, and health-related characteristics between the study sample and the general population. However, this method may not fully account for all differences between Internet survey participants and those in a nationwide representative survey, limiting our findings' generalizability. To further mitigate this limitation, harmonizing our data with a major national, representative cross-sectional study could allow us to pool data and adjust for the factor of "being an internet survey respondent," as has been done in other JACSIS studies [43].

The second limitation is related to the cross-sectional nature of the survey. This design does not allow us to establish causal relationships between the presence or absence of non-consensual sex and factors such as economic status or suicidal ideation [44].

The third limitation concerns the definition of “non-consensual sex." We did not explicitly seek a specific definition of non-consensual sexual activity, which may vary by generation or community cultures (e.g., workplace community, religious community). Sex education in Japan is known to be underdeveloped. Therefore, respondents may not necessarily share a relatively standardized understanding of this concept. We did inquire about employment status, but we did not specifically ask about non-consensual sexual activity in the workplace. However, we observed a significant correlation between being employed and experiencing non-consensual sexual activity. Also, we did not inquire about the place of experienced non-consensual sex, such as the workplace. We observed a significant correlation between employment and the occurrence of non-consensual sexual activity. However, it was not possible to ascertain whether non-consensual sex had occurred in the workplace.

Fourth, as mentioned in the section on “Analysis Subjects," our questionnaire only asked whether respondents self-identified as male or female. Therefore, among those who self-identified as female, there is a possibility that transgender women or individuals who identify as bigender may also be included. This follows the convention in Japan, where many national surveys still only inquire about male or female gender identities.

## Conclusions

This study underscores the critical need for mental and financial support for young women, highlighting the importance of early intervention for economically vulnerable groups. Comprehensive education on sexual consent is essential, especially during societal upheavals like the COVID-19 pandemic, to prevent non-consensual sex and support affected individuals.

The findings suggest key directions for public health strategies and policies. Integrating mental health and financial support services for economically disadvantaged youth is essential. Additionally, incorporating sexual consent education into school curricula and public health campaigns can help reduce non-consensual sex. Strengthening support networks during emergencies can enhance community resilience against future public health crises.
